# Design of potent fluoro-substituted chalcones as antimicrobial agents

**DOI:** 10.1080/14756366.2016.1265517

**Published:** 2017-01-24

**Authors:** Serdar Burmaoglu, Oztekin Algul, Arzu Gobek, Derya Aktas Anil, Mahmut Ulger, Busra Gul Erturk, Engin Kaplan, Aylin Dogen, Gönül Aslan

**Affiliations:** a Tercan Vocational High School, Erzincan University, Erzincan, Turkey;; b Department of Chemistry, Faculty of Science, Ataturk University, Erzurum, Turkey;; c Department of Pharmaceutical Chemistry, Faculty of Pharmacy, Mersin University, Mersin, Turkey;; d Department of Pharmaceutical Microbiology, Faculty of Pharmacy, Mersin University, Mersin, Turkey;; e Advanced Technology Education, Research, and Application Center, Mersin University, Mersin, Turkey;; f Department of Medical Microbiology, Faculty of Medicine, Mersin University, Mersin, Turkey

**Keywords:** Chalcone, antitubercular activity, antimicrobial activity, SAR

## Abstract

Owing to ever-increasing bacterial and fungal drug resistance, we attempted to develop novel antitubercular and antimicrobial agents. For this purpose, we developed some new fluorine-substituted chalcone analogs (**3**, **4**, **9–15**, and **20–23**) using a structure–activity relationship approach. Target compounds were evaluated for their antitubercular efficacy against *Mycobacterium tuberculosis* H37Rv and antimicrobial activity against five common pathogenic bacterial and three common fungal strains. Three derivatives (**3**, **9**, and **10**) displayed significant antitubercular activity with IC_50_ values of ≤16,760. Compounds derived from trimethoxy substituent scaffolds with monofluoro substitution on the B ring of the chalcone structure exhibited superior inhibition activity compared to corresponding hydroxy analogs. In terms of antimicrobial activity, most compounds (**3**, **9**, **12**–**14**, and **23**) exhibited moderate to potent activity against the bacteria, and the antifungal activities of compounds **3**, **13**, **15**, **20**, and **22** were comparable to those of reference drugs ampicillin and fluconazole.

## Introduction

Tuberculosis, caused by *Mycobacterium tuberculosis*, is one of the most important diseases worldwide. According to a 2014 report by the WHO, incidences, prevalence, and mortality rates of tuberculosis have globally decreased. However, in 2014 alone, an estimated 9.6 million new cases of tuberculosis and 1.5 million deaths caused by the disease were reported^1^. Furthermore, the emergence of multidrug-resistant and extensively drug-resistant strains has led to a growing need for effective novel agents in the fight against tuberculosis. Unfortunately, there are very few new drugs being developed for this purpose^2^. Consequently, there is a growing need for the development of effective drugs to combat tuberculosis.

The incidences of failure in the treatment of bacterial and fungal infections have increased because of the emergence of multidrug-resistant strains due to misuse of antimicrobial drugs^3^. Therefore, the synthesis of effective, novel antimicrobial compounds has become extremely important.

Chalcones are compounds with simple chemistry that offer easy synthetic access to various substituted derivatives. As well as being important constituents of various natural products, chalcones, also called α,β-unsaturated ketones, are important synthetic precursors. Chalcones and their synthetic derivatives possess extensive pharmacological properties, such as antihypertensive, antiplatelet, antidiabetic, antineoplastic, antiangiogenic, antiretroviral, anti-inflammatory, antihistaminic, antioxidant, antitubercular, antifungal, anti-invasive, and antiulcer properties^4^. Chalcones can be easily synthesized via the Claisen–Schmidt reaction of acetophenones and benzaldehydes under basic conditions.

In recent years, compounds containing fluorine have become common as potential lead drugs^5^. It has been reported that insertion of a fluorine atom into a biologically active compound results in minimal steric change, thus maintaining interactions with enzyme active sites, receptor recognitions sites, and other biological systems^6^. Furthermore, it has been indicated that the high electronegativity of fluorine can lead to significant changes in the physical and chemical properties of the molecule^6^^,^^7^. For example, it is known that a moderate to high change in the lipophilicity of a compound can impart it with improved antitubercular activity. It has been established in recent literature that fluorine-substituted compounds exhibit improved bioactivity and efficacy. Compounds with a chalcone backbone have also been reported to exhibit antimicrobial activity^8–10^.

In this study, we performed antitubercular and antimicrobial activity studies on eleven B-ring fluoro-substituted chalcones and two nonsubstituted chalcones that we have previously synthesized in an efficient, high-yielding manner^11^. These activity studies indicated that they may be used in the treatment of tuberculosis and other bacterial and fungal infections.

### Experimental

#### General

All reagents used were commercially available unless otherwise specified and all solvents were distilled before use. Melting points were measured with Gallenkamp melting point devices. IR Spectra: PerkinElmer Spectrum One FT-IR spectrometer. ^1^H- and ^13^C-NMR Spectra: Varian 400 and Bruker 400 spectrometers. Elemental analysis results were obtained on a Leco CHNS-932 instrument.

#### Synthesis

(*E*)-3-(4-Fluorophenyl)-1-(2,4,6-trimethoxyphenyl)prop-2-en-1-one (**11**);
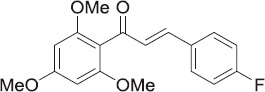


To a solution of 2,4,6-trimethoxyacetophenon (**1**) (1 g, 4.75 mmol) in MeOH (20 mL) 4-fluorobenzaldhyde (**7**) (0.6 mL 7.6 mmol) and 50% KOH solution (10 mL) was added sequentially and stirred for 15 h at room temperature. After 15 h, solvent was evaporated; 2 M HCl solution (15 mL) was added and crude product was extracted with DCM (3 × 20 mL). The combined extracts were dried over Na_2_SO_4_. The solvent was removed *in vacuo* and the remaining residue purified via coloumn chromatography over silica gel using gradient elution with EtOAc and Hexanes to yield compound **11**, as a yellow solid (80% yield). Rf (EtOAc/Hexanes 30:70) = 0.27; MP = 122–123 °C; IR (KBr, cm^−^ ^1^) *v*_max_ 3502, 2941, 2841, 1651, 1599; Anal. calcd for C_18_H_18_O_4_: C, 68.35; H, 5.42; Found: C, 68.16; H, 5.38.

^1^H NMR (400 MHz, CDCl_3_) δ 7.52–7.48 (*m*, 2H), 7.32 (d, 1H, B part of AB system, *J* = 16 Hz), 7.07–7.01 (*m*, 2H), 6.87 (d, 1H, A part of AB system, *J* = 16 Hz), 6.15 (*s*, 2H), 3.84 (*s*, 3H), 3.76 (*s*, 6H).

^13^C NMR (100 MHz, CDCl_3_) δ 194.1, 164.0 (d, C-20, *J*_CF _=_ _249.8 Hz), 162.7, 159.1, 142.8, 131.5, 130.4 (d, C-18, *J*_CF _=_ _8.4 Hz), 129.0, 116.1 (d, C-19, *J*_CF _=_ _21.7 Hz), 111.9, 90.9, 56.1, 55.7.

#### Antitubercular studies

Agar proportion method: The MIC values of each synthesized compound **3, 4, 9–15, 20–23** were obtained by agar dilution in duplicate as recommended by the Clinical Laboratory Standards Institute (CLSI)^12^^,^^13^. Positive and negative growth controls were used in each assay. INH (Sigma I3377) and EMB (Sigma E4630) were used as control agents. *M. tuberculosis* H37Rv was used as the standard strain and was provided by Refik Saydam National Public Health Agency, National Tuberculosis Reference Laboratory, Ankara, Turkey. Stock solutions of the tested compounds and reference compounds were prepared in DMSO/H_2_O (50%) at a concentration of 1000 µg/mL. These solutions were then filtered through a 0.22-µm membrane filter (Millex-GP SLGP033RS, Merck Millipore, Merck KGaA Darmstadt, Germany). Middlebrook 7H10 agar medium (BBL, Becton Dickinson and Company, Sparks, MD) was supplemented with oleic acid–albumin–dextrose–catalase (OADC, BBL, Becton Dickinson and Company, Sparks, MD). Compounds and control agents were added to obtain an appropriate final concentration in the medium. The final concentrations of INH and EMB were 0.2–1 µg/mL and 5–10 µg/mL, respectively. Compounds were prepared at final concentrations of 5, 10, 20, 40, and 80 µg/mL. Agar without any reference or tested compounds was used as a positive growth control, and 3.0 mL of prepared medium was dispensed into sterile tubes. The DMSO concentration in the final solutions was not above 1% for antimycobacterial activity.

**Figure 1.**
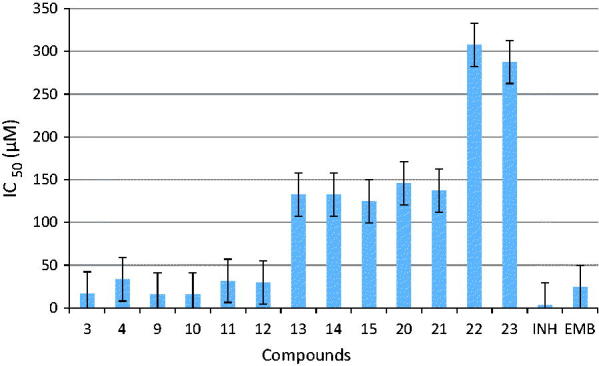
Comparative analysis of the antitubercular activity of the synthesized compounds and the standard drugs EMB and INH.

Inoculum preparation: H37Rv was maintained in Lowenstein–Jensen medium. A culture suspension was prepared by subculturing in Middlebrook 7H9 broth (BBL, Becton Dickinson and Company, Sparks, MD) supplemented with 10% OADC at 37 °C for 7–10 days, until a density corresponding to 10^–2^ to 10^–4^ dilutions were obtained from McFarland standard No. 1. Then, 0.1 mL of the diluted suspension was inoculated onto the control and the other tubes with compounds in different concentrations. The tubes were incubated at 37 °C in a 5% CO_2_ atmosphere for 3 weeks. The MIC values were defined as the lowest concentration that inhibited more than 90% of the bacterial growth, and the results for INH and EMB were interpreted according to the CLSI. The MIC was considered as the lowest concentration that showed no visible colonies in all dilutions. The biological activity studies of the compounds were performed twice. The results of these two studies were almost the same. The IC_50_ values were calculated as the average activity values for the compounds. The results are given in Figure 1.

#### Antimicrobial activity studies

Antimicrobial susceptibility testing was performed by the modification of literature methods^14^^,^^15^. We used the microbial strains *Staphylococcus aureus* (ATCC 25925), *Streptococcus pyogenes* (ATCC 19615), *Enterococcus faecalis (ATTC 29212), Escherichia coli* (ATCC 25293), *Pseudomonas aeruginosa* (ATCC 27853), *Candida albicans* (ATCC 10231), *Candida glabrata* (RSHM 40199), and *Candida parapsilosis* (ATTC 22019).

The fungal and bacterial cell inoculum were prepared from a stock culture grown in tryptic soy agar (TSA) at 28 °C for 24 h, and Mueller–Hinton agar (MHA) at 37 °C for 24 h, respectively. The microorganism suspension concentrations were adjusted according to McFarland 0.5 turbidity tubes using sterilized saline. Stock solutions of the title compounds were prepared in DMSO at 1000 µg/mL. A modified microdilution test was applied for antimicrobial activity, and the experiments were run in duplicate independently.

For antifungal activity testing, 100 µL Tryptic Soy Broth (TSB) was added to each of the 11 wells. A 100 µL aliquot of the tested chemical solution was added to the first well, and twofold dilutions were prepared. Then, 5 µL of fungal suspension was added to each tube except the last one, which acted as the control well.

For antibacterial activity testing, 100 µL Mueller–Hinton broth (MHB) was added to each of the 11 wells. A 100 µL aliquot of the chemical derivative solution was added to the first tube, and twofold dilutions were prepared. Then, 5 µL of the bacterial suspension was added to each tube, except the last control well. A control tube containing 5 µL of the fungal and bacterial suspensions alone without the tested compounds was also prepared. All plates were incubated at 28 °C (for fungi) and at 37 °C (for bacteria) for 24 h. After incubation, the MICs (Tables 2 and 3) were obtained by noting the growth inhibitions. The concentration resulting in a 50% reduction in the optical density (OD) values was compared to a reproduction control at 450 nm by spectrophotometric evaluation and defined as the MIC value. Fluconazole and ampicillin were used as reference drugs. The results were read visually and by measuring optical density for 24 h.

## Result and discussion

### Chemistry

**Scheme 1.**
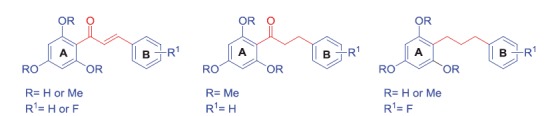
General structures of synthesized compounds.

We previously reported the synthesis of 11 B-ring fluoro-substituted and two nonsubstituted chalcone derivatives^11^. The general structure of synthesized compounds is shown in Scheme 1.

**Scheme 2.**
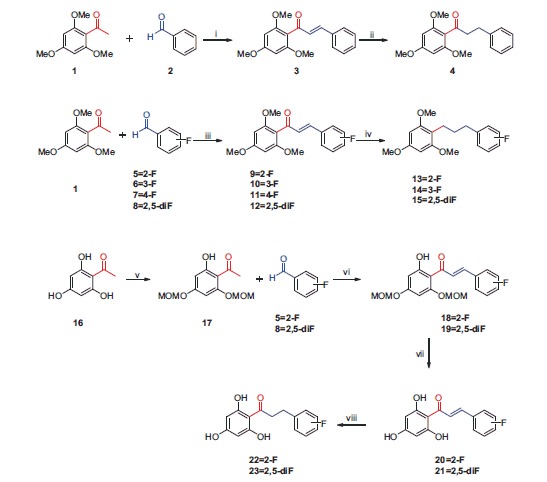
Preparation of non- and fluoro-substituted trimethoxy/trihydroxy chalcones. Reagents and conditions: (i) 50% KOH, MeOH, (ii) H_2_, Pd-C, EtOH, (iii) 50% KOH, MeOH, (iv) H_2_, Pd-C, EtOH, (v) DIPEA, MOMCl, DCM, (vi) 50% KOH, MeOH, (vii) 12M HCl, EtOAc, (viii) H_2_, Pd-C, EtOH.

Using this procedure^11^, base-catalyzed Claisen–Schmidt condensation afforded chalcones **3, 9–11,** and **12** in yields of 80–95%. Hydrogenation of the chalcone compounds over Pd–C gave **4**, **13**, **14,** and **15** in yields of 70–90%. Trihydroxy-chalcones were synthesized using the same procedure. Before the condensation reaction, the methoxymethyl ether (MOM) group was used to protect the hydroxyl groups. The base-catalyzed reaction was then used to afford MOM-protected chalcones. After deprotection, hydrogenation of **20** and **21** over Pd–C gave the target compounds **22** and **23** in yields of 80% and 85%, respectively. The general method for the synthesis of these chalcones is briefly described in Scheme 2.

All compounds except **11** were previously synthesized by our group^11^. We report here the synthesis of compound **11** and its structural details.

Modification of compound **3** (the methoxy derivative with non fluoro substituent) for activity modulation was made through preparation of compounds with fluoro substituents on the B ring only (**9, 10, 11,** and **12**), and with hydroxyl substituents on the A ring and fluoro substituents on the B ring (**20** and **21**). Versions of these compounds with semisaturated or saturated linkers were also prepared (**4, 13–15, 22,** and **23**). The synthesized compounds were fully characterized with common spectroscopy techniques.

To evaluate the potential of the basic pharmacophore of chalcone, **3** was evaluated for its antimicrobial efficacy against the *Mycobacterium tuberculosis* H37Rv strain and several other bacteria and fungi. The different functional groups carry on aromatic rings of chalcones are responsible for their antimicrobial activities. The details of the substitution patterns in the target compounds are presented in Table 1.

**Table 1.** Substitution patterns and antitubercular activity of target compounds (**3, 4, 9–15,** and **20–23**).


Compound	Main structure	R	R^1^	cLog *p*^a^
**3**	C	Me	H	3.21
**4**	D	Me	H	3.23
**9**	C	Me	2-F	3.36
**10**	C	Me	3-F	3.36
**11**	C	Me	4-F	3.36
**12**	C	Me	2,5-di F	3.52
**13**	E	Me	2-F	4.74
**14**	E	Me	3-F	4.74
**15**	E	Me	2,5-di F	4.90
**20**	C	H	2-F	2.57
**21**	C	H	2,5-di F	2.73
**22**	E	H	2-F	2.59
**23**	E	H	2,5-di F	2.75

a cLog *p* value of the synthesized compounds calculated using ChemBioDrawUltra 12.0.3.

**Table 2.** MICs of compounds **3, 4, 9–15,** and **20–23** and the standard ampicillin against the selected bacterial strains.

	Minimal inhibitory concentration, MIC (μg/mL)				
Compounds	*S. aureus*	*S. pyogenes*	*E. faecalis*	*E. coli*	*P. aeruginosa*
****3****	31.25	31.25	31.25	31.25	62.5
****4****	62.5	31.25	62.5	31.25	31.25
****9****	62.5	62.5	31.25	62.5	62.5
****10****	125	62.5	125	125	125
****11****	62.5	62.5	62.5	125	125
****12****	15.6	62.5	31.25	62.5	62.5
****13****	62.5	62.5	31.25	31.25	62.5
****14****	62.5	62.5	31.25	62.5	62.5
****15****	125	125	62.5	125	125
****20****	125	125	125	125	125
****21****	62.5	125	125	125	125
****22****	125	125	125	125	125
****23****	7.8	62.5	125	125	125
**Ampicillin**	-	-	62.5	3.9	31.25

?: All tested concentrations are active.

**Table 3.** MICs of compounds **3, 4, 9–15, 20–23** and the standard fluconazole against selected fungi.

	Minimal inhibitory concentration, MIC (μg/mL)		
Compounds	*C. albicans*	*C. glabrata*	*C. parapsilosis*
**3**	15.62	31.25	62.5
**4**	62.5	62.5	62.5
**9**	250	250	62.5
**10**	125	62.5	62.5
**11**	62.5	62.5	62.5
**12**	125	125	62.5
**13**	125	31.25	31.25
**14**	125	125	62.5
**15**	62.5	31.25	31.25
**20**	15.62	62.5	62.5
**21**	125	125	62.5
**22**	31.25	31.25	31.25
**23**	250	125	62.5
**Fluconazole**	-	31.25	-

?: All tested concentrations are active.

### Antitubercular studies

All the target compounds (**3, 4, 9–15,** and **23**) were screened against *M. tuberculosis* H37Rv using the agar proportion method. Isoniazid (INH) and ethambutol (EMB) were used as the positive drug standards for the assay. The *in vitro* antimycobacterial activity in terms of the minimum inhibitory concentration (MIC) values of the target compounds and the standard drugs are shown in Figure 1. The three derivatives **3**, **9**, and **10** show significant activities, with IC_50_ values ≤16,760, and compounds **11** and **12** exhibit moderate activities with IC_50_ values ≤31.63. The remaining eight compounds **4, 13–15, 20–22,** and **23** exhibit very low activity. The compounds derived from trimethoxy and non- or monofluoro-substituted chalcone scaffolds exhibit superior inhibition activity to their hydroxyl- and difluoro-substituted analogs. This observation clearly indicates that the trimethoxy A ring and fluoro groups on the B ring enhance the inhibition activity of the molecules. Furthermore, these results suggest that further structural modification of the above-mentioned drug molecules (**3**, **9**, and **10**) using a structure–activity relationship (SAR) approach could be a promising strategy for the identification of new leads toward the development of potent antitubercular agents.

### Antimicrobial studies

#### Antibacterial studies

The antibacterial activities of the target compounds (**3, 4, 9–15,** and **20–23**) against the five common pathogenic bacterial strains *S. aureus*, *S. pyogenes*, *E. faecalis*, *E. coli*, and *P. aeruginosa* is shown in Table 2. The experiments were performed using the microdilution method with reference to the MIC values of the compounds. The well-known commercial antibiotic ampicillin was used as the standard drug during the assay.

Compounds **3** and **4** show significant activity against almost all the bacterial strains, and compound **23** is the most active compound against *S. aureus*. Interestingly, all the compounds derived from trimethoxy-substituted chalcone scaffolds exhibit significant inhibition activities against all the tested strains, while the other compounds exhibit moderate and low activity.

#### Antifungal studies

Compounds **3, 4, 9–15,** and **20–23** were evaluated for antifungal activity against the *C. albicans, C. glabrata*, and *C. parapsilosis* fungal strains. The MIC values obtained for the compounds are given in Table 3. Fluconazole was used as the reference for inhibitory activity against fungi. The antifungal activities of all the compounds are comparable to that of fluconazole.

Compounds **3, 13, 15, 20,** and **21** are the most active compounds, with compounds **3** and **20** exhibiting excellent activity against *C. albicans* at MICs of 15.62 µg/mL. Compounds **3, 13, 15,** and **21** exhibit very good activity against *C. glabrata* at an MIC of 31.25 µg/mL, while the other compounds (**9–13, 20, 22,** and **23**) exhibit moderated inhibition with MICs of 62.5 and 125 µg/mL, as compared to the standard fluconazole (31.25 µg/mL).

Compounds **13, 15,** and **21** exhibit the highest activity against *P. parapsilosis*, with MICs of 31.25 µg/mL, whereas all other compounds exhibit low inhibitory activity compared to that of fluconazole, with MICs of 62.5 µg/mL. Compound **21**, the hydroxyl and difluoro-substituted chalcone analog, exhibits the highest activity.

#### SAR

The SAR of compounds **3, 4, 9–15,** and **20–23** was explored using the data presented in Tables 1–3, and reveals that the presence of three methoxy groups on the A ring of the chalcone increases antitubercular activity and that activity decreases in the presence of hydroxyl groups on the A ring. Chalcone and saturated chalcone compounds exhibit more pronounced antibacterial activity.

More specifically, compounds with fluoro substituents in position 2 and/or position 5 of the B ring in the chalcone structure exhibit significantly increased potency against the microbial strains. The influence of Log *p* on the antifungal activity of the compounds appears to be important: as Log *p* increases, the antifungal activity increases. Furthermore, the use of semi-saturated linker compounds appears to improve activity against all targets.

## Conclusions

**Figure 2.**
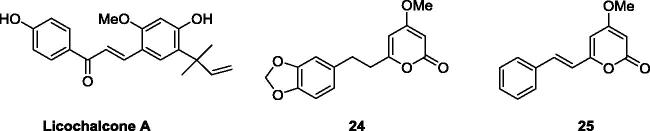
Some natural anti-tubercular agents.

Recently, chalcone-like compounds, such as licochalcone A, which are present in *Glycyrrhiza inﬂata*, and **24** and **25**, present in *Piper sanctum*, have been reported to exhibit potent antitubercular activity against *Mycobacterium tuberculosis,* with MICs of 7.1, 32, and 4.0 µg/mL, respectively (Figure 2)^16^^,^^17^.

In this study, based on the recent literature data, we designed and tested a series of 2,4,6-trimethoxy or hydroxyl and non/mono- or difluoro-substituted chalcone derivatives in order to improve the antitubercular and antimicrobial activity of previously synthesized chalcones and to gain an understanding of their SAR in the context of anti*-M*. *tuberculosis* and antimicrobial activities (Tables 1–3).

The synthetic route for chalcone derivatives (**3, 4, 9–15,** and **20–23**) is represented in Scheme 1. The 13 chalcone derivatives were prepared *via* two steps. The compounds tested for antitubercular activity were divided into two series; series A comprised trimethoxy-substituted compounds **3, 4, 9–15** derived from the parent structure **3** with fluoro substituents in position 2–5 of the B ring, and series B contained three hydroxyl substituents on the A ring and a mono- or difluoro-substituted B ring (**20–23**).

Preliminary studies revealed that compounds (**3, 9, 10**) exhibited the greatest antitubercular activity with IC_50_ values ≤16,760, which are comparable to the antitubercular drugs INH and EMB used as controls.

Modification of compound **3** for activity modulation was made by introducing fluorine atoms or saturation. These compounds were found to be either far less active than **3** or inactive. It is widely known that molecular ﬂexibility plays an important role in drug–protein interactions. However, we found that the conformationally restricted chalcones were more active than the other chalcones. This modification was made in such a way that the total lipophilic nature of the compounds was similar (The Log *p* values for compounds are shown in Table 1). We also attempted to assess the importance of the structure of the chalcone compounds to their effectiveness as antimicrobial agents.

The synthesized compounds were also evaluated for their *in vitro* antimicrobial activity against several bacterial and fungal strains. The tested compounds were inactive against bacteria and fungi, and they did not exhibit significant antibacterial and antifungal activities, except against *S. aureus*, in comparison to ampicillin and fluconazole. However, the chalcone analogs exhibited considerable antimicrobial activity. Interestingly, compounds **3** and **4** exhibited wider activity than the other compounds. Compounds **3, 20,** and **11, 23** exhibited higher antibacterial and antifungal activity against *S. aureus* and *C. albicans,* respectively.

In conclusion, assessment of the biological activity of these molecules indicated that conformationally restricted chalcones are superior to their straight chain analogs, possibly due to molecular ﬂexibility, and may be good leads for the future development of antitubercular drugs.

## Supplementary Material

IENZ_1265517_Supplementary_Material.pdf
