# Supplementary material for: Design of potent fluoro-substituted chalcones as antimicrobial agents
Source: J Enzyme Inhib Med Chem. 2017 Jan 24;32(1):490–5. doi: 10.1080/14756366.2016.1265517 (PMC6010113; doi:10.1080/14756366.2016.1265517)
Supplement: IENZ_1265517_Supplementary_Material.pdf [file IENZ_A_1265517_SM2531.pdf]

SUPPORTING INFORMATION

Design of potent fluoro-substituted chalcones as antimicrobial agents

Serdar Burmaoglu[a,c]*, Oztekin Algul[b]*, Arzu Gobek[c], Derya Aktas Anıl[c], Mahmut Ulger[d], Busra Gul Erturk[b], Engin Kaplan[e], Aylin Dogen[d], Gönül Aslan[f]

[a]Tercan Vocational High School, Erzincan University, 24800, Erzincan, Turkey
[b]Department of Pharmaceutical Chemistry, Faculty of Pharmacy, Mersin University, 33169, Mersin, Turkey
[c]Department of Chemistry, Faculty of Science, Ataturk University, 25240, Erzurum, Turkey
[d]Department of Pharmaceutical Microbiology, Faculty of Pharmacy, Mersin University, 33169, Mersin, Turkey
[e]Advanced Technology Education, Research, and Application Center, Mersin University, 33343, Mersin, Turkey
[f]Department of Medical Microbiology, Faculty of Medicine, Mersin University, 3343, Mersin, Turkey

* These authors contributed equally.

Contents

Chemistry experimental procedure:……………………………………………………………2

Copies of ^{1}H and ^{13}C NMR spectra of compound **11**……..2

∗ Corresponding authors. These authors contributed equally. Tel.: +90-324-341-2815; fax: +90-324-341-3022 (O.A); +90- 446-441-3627; fax: +90-446-441-3672 (S.B) e-mail: oztekinalgul@mersin.edu.tr, sburmaoglu@erzincan.edu.tr

(E)-3-(4-fluorophenyl)-1-(2,4,6-trimethoxyphenyl)prop-2-en-1-one (11);

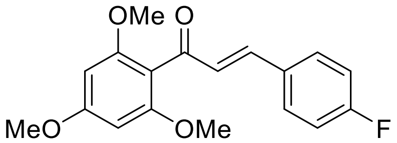

To a solution of 2,4,6-trimethoxyacetophenon (**1**) (1g ,4.75 mmol) in MeOH (20 mL) 4-F benzaldehyde (**7**) (0.6mL 7.6 mmol) and 50% KOH solution (10 mL) was added sequentially and stirred for 15 h at room temperature. After 15 h solvent was evaporated. 2M HCl solution (15 mL) was added and crude product was extracted with DCM (3x20 mL). The combined extracts were dried over Na_2SO_4. The solvent was removed in vacuo and the remaining residue purified via coloumn chromatography over silica gel using gradient elution with EtOAc and Hexanes to yield compound **11**, as a yellow solid (80% yield). Rf (EtOAc/Hexanes 30:70) = 0.27; MP = 122-123°C; IR (KBr, cm^{-1}) vmax 3502, 2941, 2841, 1651, 1599; Anal. calcd for $C_{18}H_{18}O_4$: C, 68.35; H, 5.42; Found: C, 68,16; H, 5.38

^{1}H NMR (400 MHz, $CDCl_3$) δ 7.52–7.48 (m, 2H), 7.32 (d, 1H, B part of AB system, J = 16 Hz,), 7.07–7.01 (m, 2H), 6.87 (d, 1H, A part of AB system, J = 16 Hz,), 6.15 (s, 2H), 3.84 (s, 3H), 3.76 (s, 6H).

^{13}C NMR (100 MHz, $CDCl_3$) δ 194.1, 164.0 (d, C-20, J_{CF}=249.8 Hz), 162.7, 159.1, 142.8, 131.5, 130.4 (d, C-18, J_{CF}=8.4 Hz), 129.0, 116.1 (d, C-19, J_{CF}=21.7 Hz), 111.9, 90.9, 56.1, 55.7.

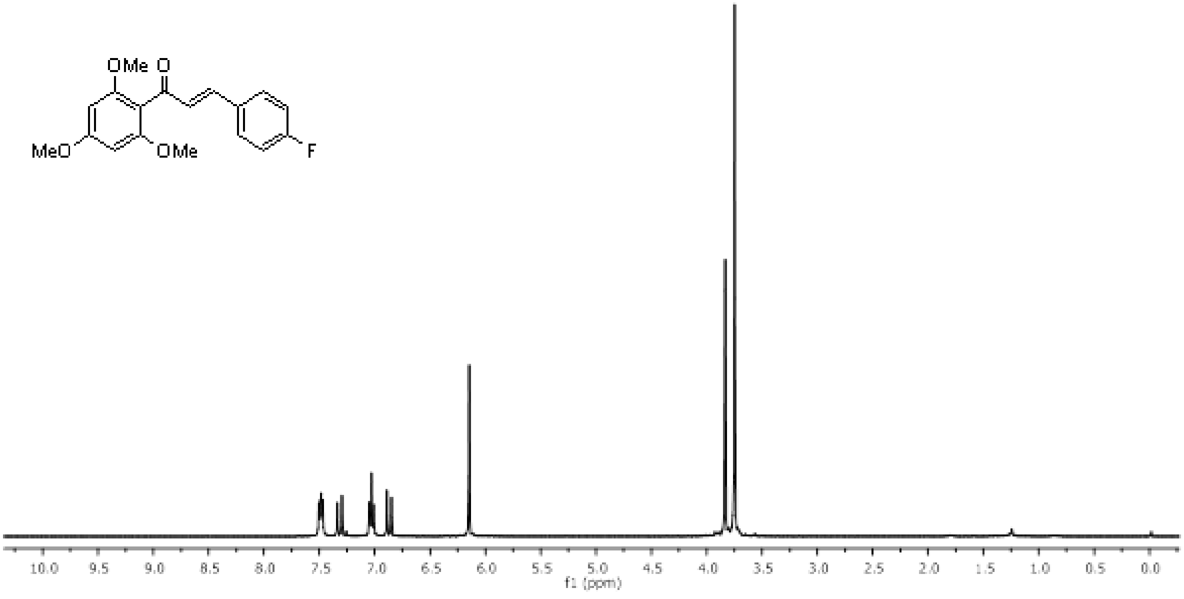

^{1}H NMR spectrum of compound **11**.

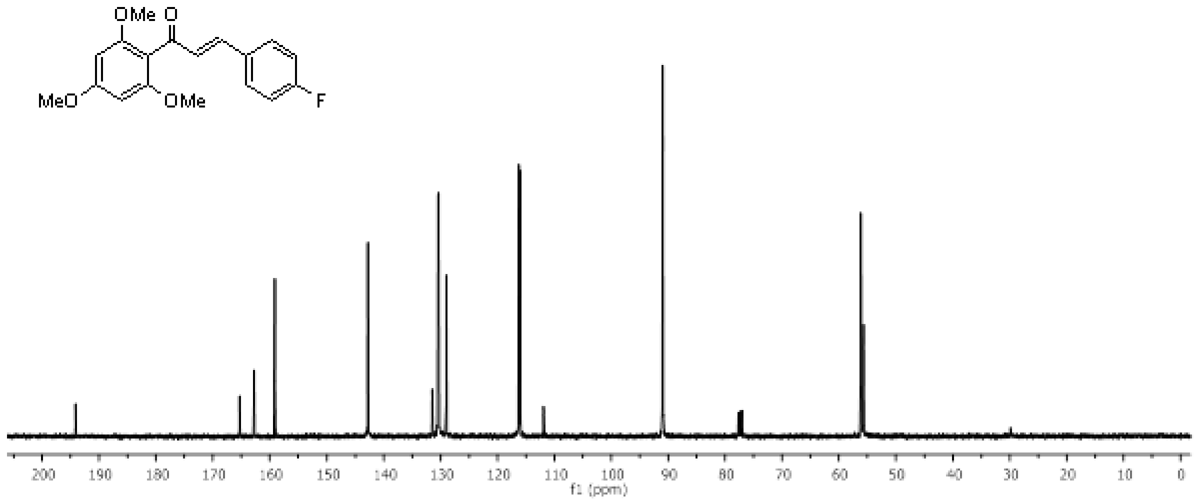

^{13}C NMR spectrum of compound **11**.
